# Relationship Between Rumen Microbial Composition and Fibrolytic Isozyme Activity During the Biodegradation of Rice Straw Powder Using Rumen Fluid

**DOI:** 10.1264/jsme2.ME23041

**Published:** 2023-09-27

**Authors:** Shuhei Takizawa, Ryoki Asano, Kenichi Abe, Yasuhiro Fukuda, Yasunori Baba, Riku Sakurai, Chika Tada, Yutaka Nakai

**Affiliations:** 1 Laboratory of Sustainable Animal Environment, Graduate School of Agricultural Science, Tohoku University, Yomogida 232–3, Naruko-onsen, Osaki, Miyagi 989–6711, Japan; 2 Research Fellow of Japan Society for the Promotion of Science, Japan Society for the Promotion of Science, 5–3–1 Kojimachi, Chiyoda-ku, Tokyo 102–0083, Japan; 3 Department of Agro-Food Science, Faculty of Agro-Food Science, Niigata Agro-Food University, Hiranedai 2416, Tainai, Niigata 959–2702, Japan; 4 Research Institute for Bioresources and Biotechnology, Ishikawa Prefectural University, Suematsu 1–308, Nonoichi, Ishikawa 921–8836, Japan

**Keywords:** biodegradation, fibrolytic isozyme, rice straw, rumen, microbial community

## Abstract

Rumen fibrolytic microorganisms have been used to increase the rate of lignocellulosic biomass biodegradation; however, the microbial and isozymatic characteristics of biodegradation remain unclear. Therefore, the present study investigated the relationship between rumen microorganisms and fibrolytic isozymes associated with lignocellulosic biomass hydrolysis. Rice straw, a widely available agricultural byproduct, was ground and used as a substrate. The biodegradation of rice straw powder was performed anaerobically in rumen fluid for 48 h. The results obtained revealed that 31.6 and 23.3% of cellulose and hemicellulose, respectively, were degraded. The total concentration of volatile fatty acids showed a 1.8-fold increase (from 85.4 to 151.6‍ ‍mM) in 48 h, and 1,230.1‍ ‍mL L^–1^ of CO_2_ and 523.5‍ ‍mL L^–1^ of CH_4_ were produced. The major isozymes identified by zymograms during the first 12‍ ‍h were 51- and 140-kDa carboxymethyl cellulases (CMCases) and 23- and 57-kDa xylanases. The band densities of 37-, 53-, and 58-kDa CMCases and 38-, 44-, and 130-kDa xylanases increased from 24 to 36 h. A microbial ana­lysis indicated that the relative abundances of *Prevotella*, *Fibrobacter*, and Bacteroidales RF16 bacteria, *Neocallimastix* and *Cyllamyces* fungi, and *Dasytricha* and *Polyplastron* protozoa were related to fibrolytic isozyme activity. The present results provide novel insights into the relationships between fibrolytic isozymes and rumen microorganisms during lignocellulose biodegradation.

Ruminants have an evolved forestomach, called the rumen, to digest ingested feed. A large volume of fluid exists in the rumen and it hosts a complex microbial community comprising 10^10^ bacterial cells, 10^4^ fungal cells, and 10^6^ protozoal cells mL^–1^ (Castillo-González *et al.*, 2014). These rumen microorganisms produce fibrolytic enzymes, such as endoglucanase (EC 3.2.1.4), exoglucanase (EC 3.2.1.91), xylanase (EC 3.2.1.8), and lignin peroxidase (EC 1.11.1.14).

Specifically, rumen bacteria secrete numerous fibrolytic enzymes into the rumen fluid or onto their membrane, and are recognized as the dominant polysaccharide-degrading group because the majority of non-rRNAs related to polysaccharide degradation are derived from bacteria (Dai *et al.*, 2015). Protozoa express fibrolytic enzymes, such as glycosyl hydrolases, polysaccharide lyases and deacetylases, xylanases, and enzymes active against pectin, mannan, and chitin (Williams *et al.*, 2020), which degrade feed particles in their vacuoles. Anaerobic fungi and several bacteria produce a number of esterases that are capable of degrading phenolic compounds and extracellular multi-enzyme complexes (cellulosomes) that tether cellulases and related accessory enzymes together, which increase fibrolytic activity by promoting synergy between individual biomass-degrading enzymes (Hess *et al.*, 2020). Fibrolytic enzymes produced by rumen microorganisms degrade fibrous materials into oligosaccharides and monosaccharides. These saccharides are converted into volatile fatty acids (VFAs), which are absorbed by host ruminants and are critical nutrients for livestock production.

Baba *et al.* (2013) focused on the fibrolytic activity of rumen microorganisms and the biodegradation of lignocellulosic biomass into biogas, bioethanol, and VFAs. Lignocellulosic biomass is mainly composed of cellulose, hemicellulose, and lignin, which bind to each other and form a strong structure that resists disintegration and hydrolysis during anaerobic digestion (Sawatdeenarunat *et al.*, 2015). Rice straw is a major lignocellulosic byproduct of rice production, and between 370 and 520×10^6^ tons of rice straw are produced annually worldwide (Hung *et al.*, 2020). However, rice straw is often dumped or burned in rice fields. A biodegradation system using rumen fluid from a‍ ‍slaughterhouse improved the degradability of grass clippings (65.9% of cellulose and 60.2% of hemicellulose degraded) (Wang *et al.*, 2018), paper sludge (7.3% of neutral detergent fiber, 7.5% of acid detergent fiber, and 19.2% of acid detergent lignin degraded) (Takizawa *et al.*, 2018), and rice straw (47.8% of cellulose and 58.9% of hemicellulose degraded) (Zhang *et al.*, 2016). Therefore, a more detailed understanding of the mechanisms underlying lignocellulose degradation is crucial for improving the bioconversion efficiency of rice straw with rumen fluid, with potential for large-scale applications.

Rumen microorganisms and fibrolytic enzymes play important roles in the biodegradation of lignocellulosic biomass via rumen fluid and may be utilized to develop biodegradation systems using rumen fluid. Previous studies characterized rumen microbial communities and their crude enzyme activities (Baba *et al.*, 2017; Li *et al.*, 2017; Liang *et al.*, 2021). We also identified several key microorganisms and fibrolytic isozymes associated with carbohydrate polymer hydrolysis by biodegrading model fibrous compounds in rumen fluid (Takizawa *et al.*, 2020b, 2022). Although the mechanisms underlying the hydrolysis of model compounds have been elucidated, the rumen microorganisms and fibrolytic isozymes associated with the biodegradation of lignocellulosic biomass (which comprises several carbohydrate polymers that form a complex structure) remain unclear.

We hypothesized that various fibrolytic microorganisms and isozymes are associated with the biodegradation of lignocellulosic biomass and hydrolysis of carbohydrate polymers. Therefore, the present study attempted to characterize the isozymatic and microbial features of lignocellulosic biomass biodegradation and elucidate the relationships between rumen microorganisms and fibrolytic isozymes associated this hydrolytic process.

## Materials and Methods

### Biodegradation of rice straw powder with rumen fluid

The present study was performed in accordance with the guidelines and regulations of Tohoku University (Miyagi, Japan). All experiments were approved by and performed in accordance with the regulations of the Institutional Animal Care and Use Committee of Tohoku University (approval number: 2019AgA-033-02). All methods were conducted in compliance with the Animal Research: Reporting of In Vivo Experiments (ARRIVE) guidelines.

Rumen fluid was collected 2‍ ‍h post-feeding using a stomach tube from a lactating Holstein cow. Cows were fed a diet consisting of 64% timothy grass and 36% concentrate and given water *ad libitum*. Collected rumen fluid was filtered through a 1×1‍ ‍mm mesh to remove coarse solids. The total solid content of filtered rumen fluid was 7.1‍ ‍g‍ ‍L^–1^.

Rice straw was collected from rice fields with permission from the Field Science Center, Graduate School of Agricultural Science, Tohoku University (Miyagi, Japan). Straw was dried at 60°C overnight, chopped into lengths of 1–2‍ ‍cm using scissors, and then ground in a laboratory mill (Osaka Chemical) for approximately 5‍ ‍min. The powder was sieved through a 1×1‍ ‍mm mesh. This powder consisted of 34.7% cellulose, 24.1% hemicellulose, 4.4% lignin, and 36.8% of other constituents.

Four grams of the rice straw powder was mixed with 200‍ ‍mL of rumen fluid and purged with nitrogen gas to remove oxygen. Buffers (such as artificial saliva) were not used in the present study because the cost of reagents is a feasible barrier to the large-scale operation of rumen fluid bioconversion systems. The biodegradation of rice straw powder was performed in a 250-mL reactor equipped with an aluminum gas bag at 37°C on a rotary shaker at 170‍ ‍rpm for 0, 12, 24, 36, and 48 h. After biodegradation, 1‍ ‍mL of samples was collected using a syringe and immediately used for protein extraction, and the residue was stored at –20°C for further chemical ana­lyses and DNA extraction. The control reactor contained only rumen fluid or rice straw powder with sterile purified water instead of the rumen fluid. All incubations were performed in duplicate.

### Analysis

The contents of three polymers (cellulose, hemicellulose, and lignin), total solids, VFAs, pH, and gas production (CO_2_ and CH_4_) were measured as previously described (Takizawa *et al.*, 2018), with some modifications. VFA concentrations were assessed using high-performance liquid chromatography (Jasco) with an ion-exchange column (RSpak KC-811; Shodex) and ultraviolet detector at 445‍ ‍nm (870-UV; JASCO). The column temperature was 60°C, the eluent consisted of 90% 3‍ ‍mM perchloric acid and 10% acetonitrile, and the flow rate was 1.0‍ ‍mL‍ ‍min^–1^. CH_4_ and CO_2_ concentrations were measured using gas chromatography (GC-8A; Shimadzu) with a SHINCARBON-ST 50/80 packed column (2 m×3‍ ‍mm internal diameter; Shinwa Chemical Industries). Argon was used as the carrier gas, and injection and detection temperatures were set to 100 and 120°C, respectively. Carboxymethyl cellulase (CMCase) and xylanase activities were measured as previously described (Takizawa *et al.*, 2019).

### Amplicon sequencing

DNA was extracted from 1‍ ‍mL of rumen fluid using a FastDNA Spin Kit for Soil (MP Biomedicals) according to the manufact­urer’s instructions. DNA libraries were constructed for ampli­con sequencing based on two-step tailed PCR using primers with a barcode for the prokaryotic 16S rRNA V4 region (Caporaso *et al.*, 2011), anaerobic fungal ITS1 (Tuckwell *et al.*, 2005), and protozoal 18S rRNA (Ishaq and Wright, 2014). The constructed libraries were subjected to 300-bp paired-end sequencing on a MiSeq (Illumina) by Seibutsu Giken. Sequence processing and taxonomic ana­lyses were performed using QIIME2 ver. 2020.8 (Bolyen *et al.*, 2019). After removing the primer sequences using the cutadapt plugin (Martin, 2011), sequence processing, such as denoising, quality filtering, dereplication, chimera removal, and merging, were conducted using the DADA2 plugin (Callahan *et al.*, 2016) with the default parameters. The number of reads before and after sequence processing is shown in Table S2. The exported amplicon sequence variants (ASVs) table was rarefied to the lowest sequence depth. Non-metric multidimensional scaling was performed based on Bray–Curtis dissimilarity using the vegan package in R (versions 2.5–6). The ASVs obtained were classified using Greengenes (ver. 13_8) for bacteria, UNITE (ver. 8.2) for fungi, and SILVA (ver. 138) for protozoa.

Sequence data are available in the DNA Data Bank of Japan (DDBJ) sequence read archive under accession number DRA014579 (DRR396149–DRR396178).

### SDS-PAGE and zymograms

One milliliter of rumen fluid was centrifuged at 12,000×*g* at 4°C for 10‍ ‍min. The precipitate was washed with 1‍ ‍mL of wash buffer (EzApply 2D kit; ATTO) at 4°C and mixed with 1‍ ‍mL of 2× sample buffer (Nacalai Tesque), containing 2‍ ‍mmol L^–1^ phenylmethylsulfonyl fluoride and 1× proteinase inhibitor. Protein extraction was performed at 4°C using two rounds of 2‍ ‍min bead-beating with Lysing Matrix E tubes (MP Biomedicals), followed by centrifugation at 12,000×*g* at 4°C for 10‍ ‍min. The supernatant was collected and stored at –80°C until SDS-PAGE and zymogram ana­lyses.

SDS-PAGE and zymography were performed as previously described (Takizawa *et al.*, 2021), with some modifications. Briefly, 20‍ ‍μL of the protein extract was loaded on 8% polyacrylamide gels containing 0.15% carboxymethyl cellulose (CMC) sodium salt or 1.0% beechwood-derived xylan for the CMCase and xylanase zymograms, respectively. Electrophoresis was performed at 200‍ ‍V for 60‍ ‍min. The separated proteins were refolded in phosphate citrate buffer (20‍ ‍mM, pH 6.5) containing 1.5% β-cyclodextrin for 30‍ ‍min. CMCase and xylanase zymograms were obtained at 37°C for 1.5 and 4 h, respectively, in 30‍ ‍mM sodium acetate buffer. Gels were stained with 0.1% Congo red, and band densities were analyzed using Fiji software (Schindelin *et al.*, 2012).

## Results

### Efficiency of rice straw powder biodegradation

**Fig. 1.**
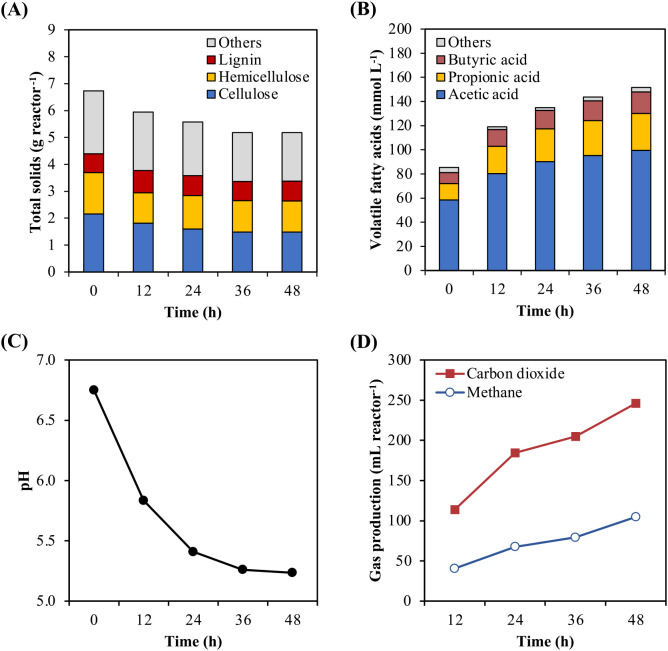
Degradation of rice straw powder during treatment with rumen fluid. (A) Total solids and polymer concentrations, (B) volatile fatty acids, (C) pH, and (D) gas production. All data represent the mean of duplicated samples.

Rice straw powder was hydrolyzed via biodegradation with rumen fluid (Fig. 1A). Total solids, cellulose, and hemicellulose contents decreased by 22.9, 31.7, and 23.6%, respectively, after 36 h. However, a decrease in lignin content was not observed. VFAs were the major metabolic products of cellulose and hemicellulose degradation (Fig. 1B). The dominant VFA was acetic acid (65.7–68.4% of total VFAs), followed by propionic and butyric acids. The total VFA concentration increased 1.8-fold in 48‍ ‍h (from 85.4 to 151.6‍ ‍mmol L^–1^), and this increase was rapid during the first 12‍ ‍h (from 85.4 to 119.0‍ ‍mmol L^–1^). Due to the accumulation of VFAs, pH declined from 6.8 to 5.3 (Fig. 1C) in 48 h. During the bioconversion of rice straw powder, 1230.1‍ ‍mL L^–1^ of CO_2_ and 523.5‍ ‍mL L^–1^ of CH_4_ were produced (Fig. 1D).

In the controls containing only rumen fluid or rice straw powder, the concentrations of metabolic products (VFAs, CO_2_, and CH_4_) were lower than those in the biodegradation of rice straw powder with rumen fluid (Fig. S1). Lactic acid was mainly detected in the control containing only rice straw powder.

### Activities of fibrolytic isozymes

**Fig. 2.**
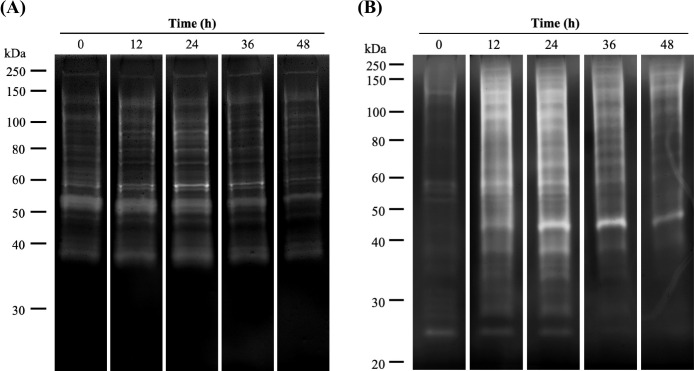
Carboxymethyl-cellulase (A) and xylanase (B) activities during the biodegradation of rice straw powder with rumen fluid. Twenty microliters of protein extract was loaded on an 8% polyacrylamide gel containing 0.15% carboxymethyl cellulose salt and 1.0% xylan from beechwood for carboxymethyl-cellulase and xylanase zymograms, respectively. Incubations for carboxymethyl-cellulase and xylanase zymograms were performed at 37°C for 90‍ ‍min and 4 h, respectively. Grouping gels were cropped from the sliced gel.

Zymography revealed shifts in CMCase activity during rice straw powder biodegradation (Fig. 2A and Table S1). Several CMCases (38–250‍ ‍kDa) were identified during biodegradation. The prevalence of 37-, 51-, 53-, 58-, and 140-kDa CMCases was high during biodegradation. The activities of 37- and 58-kDa CMCases increased by 1.70- and 4.09-fold, respectively, during the first 24 h, that of 140-kDa CMCase did not change for 48 h, and those of 51-and 53-kDa CMCases decreased after 24 h. The total band intensity of the 5 CMCases increased by 1.31-fold during the first 24‍ ‍h and then decreased by 1.70-fold. Crude CMCase activity increased from 11.31 to 14.83 unit L^–1^ during the first 12‍ ‍h and decreased after 36 h.

Zymography also revealed shifts in the activities of various xylanases during biodegradation (Fig. 2B and Table S1). A wide range of xylanases (23–250‍ ‍kDa) were identified. The prevalence of 23-, 38-, 44-, 57-, and 130-kDa xylanases was high during biodegradation. The activities of 38-, 44-, and 130-kDa xylanases increased by 14.18-, 40.37-, and 4.05-fold, respectively, during the first 24 h. The activity of‍ ‍57-kDa xylanase increased by 3.80-fold during the first 12‍ ‍h and then decreased, while that of 23-kDa xylanase decreased during biodegradation. The total band intensity of the 5 xylanases increased by 4.75-fold during the first 24‍ ‍h and then decreased by 3.02-fold. Crude xylanase activity increased from 44.93 to 49.61 unit L^–1^ during the first 12‍ ‍h and then gradually decreased.

The fibrolytic enzyme activities of the controls containing only rumen fluid or rice straw powder were compared with those in the control containing rice straw powder with rumen fluid. No changes were observed in fibrolytic enzyme activity in the control containing only rumen fluid (Fig. S2A and B), and there were no clear active bands in the control containing only rice straw powder (Fig. S2C and D).

### Rumen microbial community composition

**Fig. 3.**
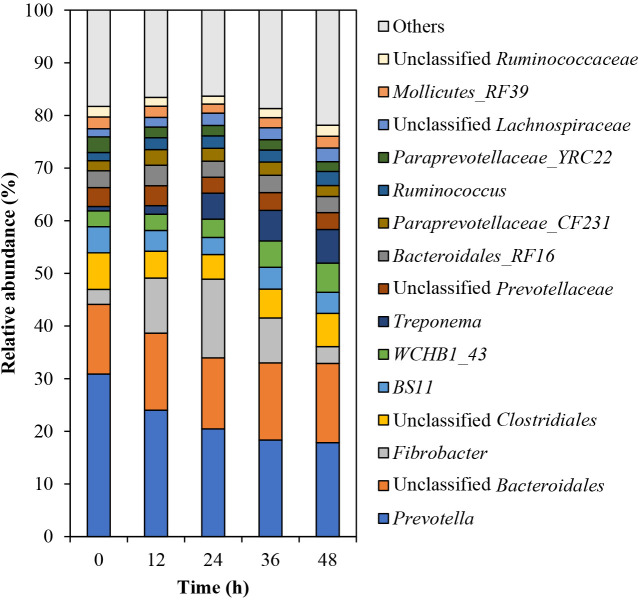
Relative abundances of rumen bacterial genera during the biodegradation of rice straw powder. The top 15 bacterial genera are shown, and all other genera are included in “others”. All data represent the mean of duplicated samples.

The bacterial community structure changed over 48 h, particularly from 0 to 12‍ ‍h (Fig. S3A). At the phylum level, *Bacteroidetes* was the most dominant throughout the treatment, followed by *Firmicutes*, *Fibrobacteres*, *Verrucomicrobia*, *Spirochaetes*, *Tenericutes*, and *Proteobacteria* (Table S3). The relative abundance of *Bacteroidetes* decreased by 13.45% over 48 h, whereas that of *Fibrobacteres* increased by 12.10% over 24 h. *Verrucomicrobia* and *Spirochaetes* also increased (by 3.00 and 5.63%, respectively) over 48 h. At the genus level, the relative abundance of *Prevotella* decreased by 13.06% over 48‍ ‍h (Fig. 3), which was similar to the decrease observed in 23-kDa xylanase activity. The relative abundance of *Fibrobacter* increased from 2.85 to 14.95% over 24‍ ‍h and then decreased to 3.20%. These dynamics of *Fibrobacter* reflected the total density of the 5 CMCase bands and 5 xylanase bands, 37- and 58-kDa CMCase activities, and 38- and 130-kDa xylanase activities. The relative abundance of Bacteroidales RF16 increased in the first 12‍ ‍h and then decreased, which was similar to the changes detected in 57‍ ‍kDa xylanase activity. The relative abundances of *Treponema*, *Ruminococcus*, and *Clostridium* increased by 5.52, 1.14, and 1.15%, respectively, over 48 h.

**Fig. 4.**
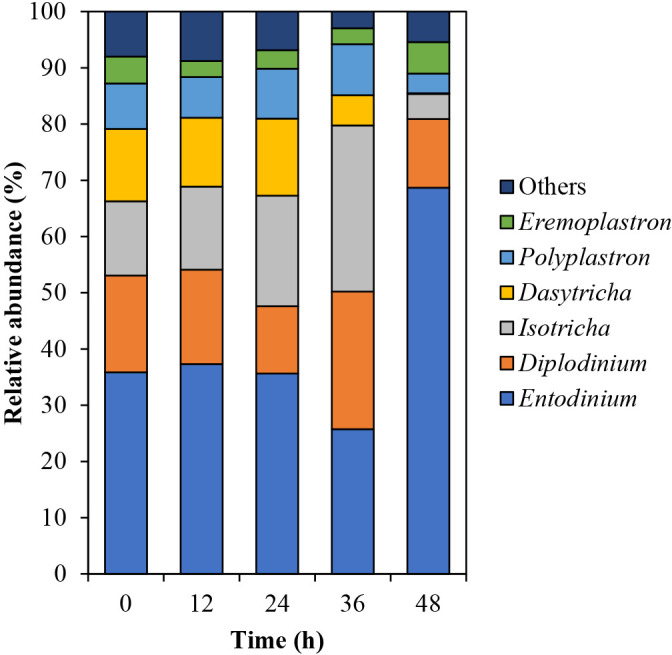
Relative abundances of rumen fungal genera during the biodegradation of rice straw powder. The top 6 fungal genera are shown, and all other genera are included in “others”. All data represent the mean of duplicated samples.

The structure of the fungal community also changed over 12‍ ‍h and was clustered at 24, 36, and 48‍ ‍h (Fig. S3B). At the phylum level, >99.99% of the sequences were classified as Neocallimastigomycota throughout the treatment (Table S3). At the genus level, *Orpinomyces* was predominant, followed by *Neocallimastix*, *Anaeromyces*, and *Caecomyces* (Fig. 4). The relative abundance of *Orpinomyces* decreased by 20.83% in 12‍ ‍h and increased to 55.41%, while that of *Neocallimastix* increased by 17.49% over 12 h, which reflected the increase in 44- and 130-kDa xylanase activities. The relative abundance of *Cyllamyces* slightly increased within 12‍ ‍h (from 0.20 to 0.21%) and decreased to 0.00%, which was similar to the change observed in 51-kDa CMCase activity.

**Fig. 5.**
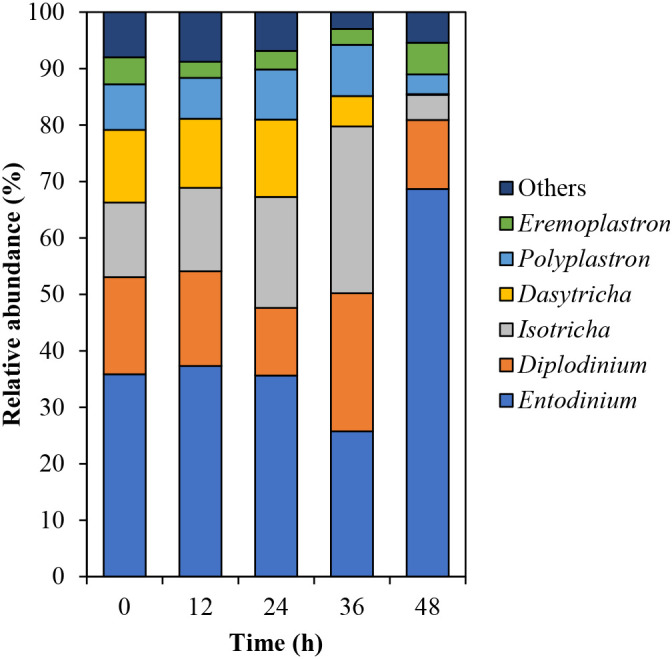
Relative abundances of rumen protozoal genera during the biodegradation of rice straw powder. The top 6 protozoal genera are shown, and all other genera are included in “others”. All data represent the mean of duplicated samples.

Differences were observed in the protozoan community structure over 48 h, with marked changes being detected from 36 to 48‍ ‍h (Fig. S3C and D). At the phylum level, more than 99.99% of sequences were classified as Ciliophora, whereas the others were unclassified (Table S3). At the genus level, *Entodinium* was the most dominant, followed by *Diplodinium*, *Isotricha*, *Dasytricha*, *Polyplastron*, and *Eremoplastron* (Fig. 5). The relative abundance of *Entodinium* decreased by 9.90% from 24 to 36‍ ‍h and increased to 68.67%, while those of *Diplodinium* and *Isotricha* increased by 12.51 and 9.87%, respectively, from 24 to 36 h. The relative abundance of *Dasytricha* and *Polyplastron* increased over 24‍ ‍h and then decreased, which was similar to the change observed in total CMCase activity.

## Discussion

We investigated the efficiency of rice straw powder biodegradation in rumen fluid. Cellulose and hemicellulose were degraded by 31.6 and 23.3%, respectively, into monosaccharides and metabolized into VFAs over 36‍ ‍h of biodegradation. However, the hydrolysis rate markedly declined from 36 to 48 h. The efficiency of polysaccharide biodegradation in the present study was higher than that reported by Bai *et al.* (2017), who evaluated the biodegradation of rice straw using *Bacillus megaterium* MYB3 for 3 days. They found that 7.11 and 22.48% of cellulose and hemicellulose, respectively, were degraded. Zhang *et al.* (2016) and Liang *et al.* (2021) performed the anaerobic fermentation of rice straw with rumen fluid and showed high cellulose and hemicellulose degradation rates (approximately 47.8 and 58.9%, respectively), which were higher than the present results. Lower efficiency in this study may be attributed to the accumulation of VFAs and decreases in pH. Moreover, the present study did not refill rumen fluid due to cost limitations; therefore, VFAs continuously accumulated during biodegradation and the pH value decreased below 5.3 after 36 h. Ruminal pH values below 5.3 induce the lysis or detachment of adherent cellulolytic bacteria, which critically inhibits cellulose digestion (Mouriño *et al.*, 2001). We also previously showed that carbohydrate polymer hydrolysis and fibrolytic enzyme activity were inhibited after decreases in ruminal pH (Takizawa *et al.*, 2020b, 2022). Therefore, we assumed that low pH values after 36‍ ‍h inhibited the production of fibrolytic enzymes by rumen microorganisms, the hydrolysis of cellulose and hemicellulose into monosaccharides, and the subsequent metabolism of monosaccharides into VFAs.

To measure fibrolytic isozyme activities during the biodegradation of rice straw powder, we investigated the activities of CMCase and xylanase isozymes using zymograms. The sum of the 5 CMCase and xylanase band densities increased during the first 24‍ ‍h and then decreased from 36 to 48 h. These changes in band densities were similar to those in crude fibrolytic enzyme activities and are consistent with cellulose and hemicellulose biodegradation rates. Wang *et al.* (2018) performed the biodegradation of lawn grass using rumen fluid for 72‍ ‍h and similarly reported increased CMCase and xylanase activities for the first 24 h, during which the contents of soluble organic compounds and VFAs also rapidly increased. These relationships between fibrolytic enzyme activities and biodegradation rates indicate that fibrolytic enzymes play an important role in cellulose and hemicellulose hydrolysis.

We also investigated changes in fibrolytic isozyme activities for 24‍ ‍h when cellulose and hemicellulose were efficiently biodegraded. The band density of 23-kDa xylanase was the highest at 0 h, but decreased as biodegradation proceeded. Similarly, 51 and 140‍ ‍kDa CMCases and 57‍ ‍kDa xylanase exhibited decreased activities after 12 h. The band densities of 37, 53, and 58‍ ‍kDa CMCases and 38, 44, and 130‍ ‍kDa xylanases increased after 24 h. These results suggest that 51- and 140-kDa CMCases and 23- and 57-kDa xylanases play important roles in the early hydrolysis of carbohydrate polymers (0–12‍ ‍h), whereas 37-, 53-, and 58-kDa CMCases and 38-, 44-, and 130-kDa xylanases are important for late hydrolysis (24–36‍ ‍h). Changes in fibrolytic isozyme activity over time were also observed in our previous study, which evaluated the biodegradation of tomato leaves with rumen fluid (Takizawa *et al.*, 2020a). Comtet-Marre *et al.* (2017) investigated the expression of genes encoding carbohydrate-active enzymes (CAZymes) in cows and found that various rumen microorganisms were involved in the production of CAZyme-encoding transcripts and the breakdown of cellulose and hemicellulose. We assumed that fibrolytic isozymes are produced by various rumen microorganisms during polysaccharide degradation, with their production varying to adapt to environmental shifts (*i.e.*, saccharide and VFA accumulation and/or decreased pH) and the surface structure of polysaccharides.

Furthermore, we compared isozymatic and microbial characteristics. In terms of bacterial genera, the relative abundance of *Fibrobacter* increased over the first 24 h, showing the same pattern as 37- and 58-kDa CMCases and 38- and 130-kDa xylanases. Changes in the relative abundances of *Prevotella* and Bacteroidales RF16 were related to 23- and 57-kDa xylanase activities, respectively. The bacterial genera *Fibrobacter* and *Prevotella* have gained much attention because they are the dominant fibrolytic bacteria in‍ ‍the rumen and are considered to play important roles in carbohydrate polymer digestion (Seshadri *et al.*, 2018). Metatranscriptomic ana­lyses of cow rumen microorganisms revealed that *Fibrobacter* and *Prevotella* contributed to 13 and 16–21%, respectively, of cellulase- and hemicellulose-encoding transcripts (Dai *et al.*, 2015). Zymogram ana­lyses also showed that six strains of *Fibrobacter succinogenes* and *Fibrobacter intestinalis* produced a wide range of CMCases and xylanases, including 37- and 58-kDa CMCases and 38- and 130-kDa xylanases (Béra-Maillet *et al.*, 2004). Matsui *et al.* (2000) performed a zymogram ana­lysis and revealed that *Prevotella ruminicola* and *Prevotella bryantii* produced a wide variety of xylanases. Although the function and enzymatic profile of Bacteroidales RF16 are unknown, this bacterial taxon was more frequently found in yaks that were fed a forage diet than in those fed a concentrate diet (Liu *et al.*, 2019), indicating that Bacteroidales RF16 is involved in the digestion of fibrous materials. We assumed that *Fibrobacter*, *Prevotella*, and Bacteroidales RF16 bacteria produce fibrolytic isozymes and play important roles in the hydrolysis of carbohydrate polymers during rice straw powder biodegradation.

Regarding the fungal genera, the relative abundance of *Neocallimastix* increased after 12 h, showing the same pattern as 44- and 130-kDa xylanase activities. In addition, *Cyllamyces* was related to the change in 51-kDa CMCase activity. Gomez de Segura and Fevre (1993) purified extracellular enzymes produced by *Neocallimastix frontalis* and identified an endoxylanase with a mole­cular mass of 45‍ ‍kDa. They also analyzed extracellular xylanases secreted by *N. frontalis* using isoelectric focusing SDS-PAGE, and identified a number of xylanases (Gomez de Segura *et al.*, 1998). However, the characteristics of the CMCases produced by *Cyllamyces* remain unclear. *Cyllamyces* may grow on a wide variety of substrates, including wheat straw and cellulose powder (Ozkose *et al.*, 2001), indicating that it produces cellulase. Furthermore, total rRNA sequencing of solid-phase rumen digesta showed that 10.48% of the total small subunit rRNA reads accounted for anaerobic fungi and mainly consisted of the genera *Neocallimastix* (56%) and *Cyllamyces* (36%) (Elekwachi *et al.*, 2017). These findings indicate that the dominant genera *Neocallimastix* and *Cyllamyces* hydrolyze carbohydrate polymers by producing fibrolytic enzymes for biodegradation.

In terms of the protozoan genera, the relative abundance of *Dasytricha* decreased after 36 h, which showed the same pattern as the activities of 51- and 53-kDa CMCases. Takenaka *et al.* (2004) analyzed the fibrolytic enzyme activity of rumen ciliates isolated from mono-faunated calves and found that *Dasytricha* did not contribute to CMCase activity. Therefore, we assumed that *Dasytricha* utilizes the metabolic products of cellulose digestion instead of producing CMCases during rice straw powder biodegradation. Our results also showed that the relative abundance of *Polyplastron* was related to the total intensity of the 5 CMCases. Béra-Maillet *et al.* (2005) isolated three rumen protozoa (*Polyplastron multivesiculatum*, *Eudiplodinium maggii*, and *Entodinium* sp.) from the rumen contents of mono-faunated sheep and found that *P. multivesiculatum* had the highest CMCase activity, producing 28- to 116-kDa CMCases. Previous metatranscriptomic ana­lyses of the rumen microbiomes of adult Holstein cows revealed that *Polyplastron* is the major cellulolytic protozoa, contributing to 20% of protozoan cellulase transcripts (Dai *et al.*, 2015). Although the characteristics of CMCases expressed by *Polyplastron* are unclear, *Polyplastron* may be involved in cellulose digestion.

Collectively, the present results indicate that fibrolytic isozyme activities are related to the dominant bacteria (*Fibrobacter*, *Prevotella*, and Bacteroidales RF16), fungi (*Neocallimastix* and *Cyllamyces*), and protozoa (*Dasytricha* and *Polyplastron*). Nine of the ten major fibrolytic isozymes were related to microbial abundance in this study; however, a relationship was not observed between relative microbial abundance and 140‍ ‍kDa CMCase activity. Previous 16S rRNA-based studies reported that approximately 77% of fiber-associated rumen microorganisms were uncultured (Koike *et al.*, 2003) and these uncultured bacteria were responsible for fiber digestion in the rumen (Koike and Kobayashi, 2009). We also suggested the importance of unclassified microorganisms in fibrolytic isozyme activity (Takizawa *et al.*, 2020b, 2022). Therefore, 140-kDa CMCase may be related to uncultured and/or minor microorganisms.

Large amounts of rice straw are produced annually worldwide, but are often dumped or burned in rice fields. We herein demonstrated that the biodegradation of rice straw powder using rumen fluid may be used to produce VFAs. Furthermore, biodegradation with rumen fluid has been shown to improve the methane yield of rice straw by 82.6% (Zhang *et al.*, 2016). Although further studies using rice straw without grinding are needed, biodegradation using rumen fluid may enable the efficient production of biomaterials and biofuels from rice straw. In addition, we assumed that increases in fibrolytic activity via the addition of fibrolytic microorganisms and enzymes may improve the degradability of rice straw. To utilize rumen microorganisms that produce optimal levels of fibrolytic enzymes, further phylogenetic ana­lyses are required at the species or strain levels using full-length 16S rRNA gene amplicon sequencing. Further investigations on amino acid sequences and enzymatic characteristics are also warranted to purify and produce fibrolytic enzymes. These efforts will enable the identification of important microorganisms and fibrolytic enzymes involved in rumen fluid-mediated rice straw biodegradation and improve the efficiency and stability of these biodegradation systems.

Overall, our chemical and zymogram ana­lyses revealed that 37-, 51-, 53-, 58-, and 140-kDa CMCases and 23-, 38-, 44-, 57-, and 130-kDa xylanases had high band densities during rice straw powder hydrolysis. The microbial ana­lysis showed that fibrolytic isozyme activity was related to the relative abundances of the dominant bacteria (*Fibrobacter*, *Prevotella*, and Bacteroidales RF16), fungi (*Neocallimastix* and *Cyllamyces*), and protozoa (*Dasytricha* and *Polyplastron*). The present results will provide a foundation for elucidating the relationships between microbial and enzymatic characteristics during the biodegradation of lignocellulosic biomass.

## Citation

Takizawa, S., Asano, R., Abe, K., Fukuda, Y., Baba, Y., Sakurai, R., et al. (2023) Relationship Between Rumen Microbial Composition and Fibrolytic Isozyme Activity During the Biodegradation of Rice Straw Powder Using Rumen Fluid. *Microbes Environ ***38**: ME23041.

https://doi.org/10.1264/jsme2.ME23041

## Supplementary Material

Supplementary Material
