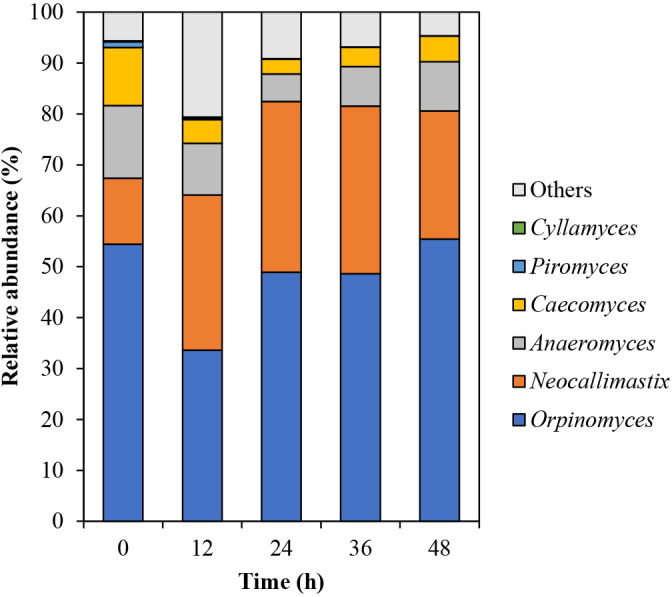# Author’s correction: Relationship Between Rumen Microbial Composition and Fibrolytic Isozyme Activity During the Biodegradation of Rice Straw Powder Using Rumen Fluid

**DOI:** 10.1264/jsme2.ME23041e

**Published:** 2024-06-05

**Authors:** Shuhei Takizawa, Ryoki Asano, Kenichi Abe, Yasuhiro Fukuda, Yasunori Baba, Riku Sakurai, Chika Tada, Yutaka Nakai

**Affiliations:** 1 Laboratory of Sustainable Animal Environment, Graduate School of Agricultural Science, Tohoku University, Yomogida 232–3, Naruko-onsen, Osaki, Miyagi 989–6711, Japan; 2 Research Fellow of Japan Society for the Promotion of Science, Japan Society for the Promotion of Science, 5–3–1 Kojimachi, Chiyoda-ku, Tokyo 102–0083, Japan; 3 Department of Agro-Food Science, Faculty of Agro-Food Science, Niigata Agro-Food University, Hiranedai 2416, Tainai, Niigata 959–2702, Japan; 4 Research Institute for Bioresources and Biotechnology, Ishikawa Prefectural University, Suematsu 1–308, Nonoichi, Ishikawa 921–8836, Japan

Vol. 38, No. 3, ME23041, 2023

 

Fig. 4

 

Incorrect


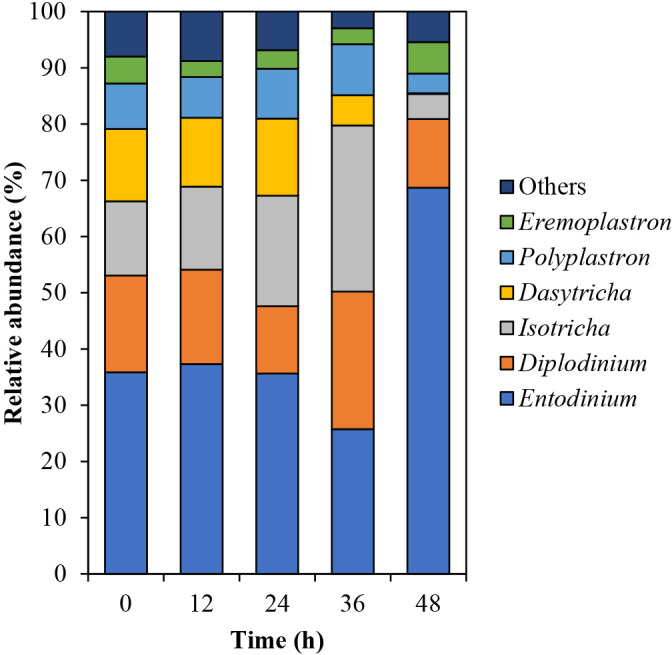


 

Correct