# Supplementary material for: Relationship Between Rumen Microbial Composition and Fibrolytic Isozyme Activity During the Biodegradation of Rice Straw Powder Using Rumen Fluid
Source: Microbes Environ. 2023 Sep 27;38(3):ME23041. doi: 10.1264/jsme2.ME23041 (PMC10522846; doi:10.1264/jsme2.ME23041)
Supplement: Supplementary file 1 — Supplementary Material [file 38_23041_s1.pdf]

Supplementary Table S1. Band strength during treatment of rice straw powder with the rumen fluid.

	Treatment (h)				
	**0**	**12**	**24**	**36**	**48**
**CMCase**					
**Band strength**					
Total	40,621	44,739	53,116	46,351	31,324
140 kDa	2,693	4,285	2,312	3,407	3,435
58 kDa	1,087	2,218	4,447	3,169	1,517
53 kDa	8,267	7,893	8,386	6,938	4,008
51 kDa	3,923	4,251	2,765	781	0
37 kDa	8,389	10,562	14,255	11,066	9,590
**Enzyme activity (Unit L^{-1})**	11.317	14.829	14.100	9.125	8.256
**Xylanase**					
**Band strength**					
Total	3,601	11,382	17,100	11,612	5,656
130 kDa	504	1,604	2,042	1,527	1,271
57 kDa	674	2,562	1,024	671	174
44 kDa	120	2,063	4,844	4,782	1,647
38 kDa	175	904	2,482	377	160
23 kDa	1,292	880	866	277	263
**Enzyme activity (Unit L^{-1})**	44.928	49.613	39.051	36.025	28.870

Top 5 of CMCases and xylanases having a high peak density were shown. Each peak density was analyzed using the gel for CMCase and xylanase zymograms (Fig. 2).

Supplementary Table S2. Details of each library before and after sequence processing.

	Input	Filtered	Denoised	Merged	Non-chimeric	Read length*
**16S rRNA V4**						
0h-1	78,364	72,604	67,505	56,423	53,031	254
0h-2	77,447	71,677	66,468	56,189	53,397	
12h-1	67,779	62,700	58,452	50,574	47,879	
12h-2	60,621	56,348	52,728	46,048	43,233	
24h-1	70,217	65,038	60,470	52,073	49,087	
24h-2	88,889	82,533	77,782	66,373	60,513	
36h-1	76,946	71,170	66,818	58,218	53,807	
36h-2	68,661	63,467	59,206	50,107	47,271	
48h-1	75,107	69,644	65,362	55,617	51,838	
48h-2	59,592	55,011	50,866	43,810	41,948	
**Anaerobic fungal ITS1**						
0h-1	73,938	64,959	64,712	59,623	57,330	236
0h-2	60,831	56,885	56,620	52,048	50,836	
12h-1	64,967	58,126	57,885	52,380	51,015	
12h-2	75,027	64,064	63,771	59,314	56,021	
24h-1	62,469	54,346	54,115	50,471	47,767	
24h-2	63,079	58,551	58,367	54,261	52,323	
36h-1	71,212	64,922	64,493	60,020	57,166	
36h-2	80,173	74,388	74,251	69,527	67,332	
48h-1	63,961	59,872	59,691	55,475	53,335	
48h-2	53,241	49,480	49,307	45,246	44,005	
**Protozoal 18S rRNA**						
0h-1	65,357	49,368	49,203	42,576	31,448	442
0h-2	71,970	55,534	55,370	51,420	37,126	
12h-1	72,929	57,763	57,588	49,617	38,143	
12h-2	62,859	49,219	49,136	42,528	30,906	
24h-1	57,318	43,983	43,927	37,790	30,585	
24h-2	51,796	42,777	42,623	38,563	29,761	
36h-1	85,931	67,243	67,115	58,024	41,621	
36h-2	77,163	63,547	63,457	58,624	40,635	
48h-1	78,303	64,921	64,868	59,900	42,301	
48h-2	79,385	59,187	59,079	54,761	40,718	

* Read length represents the mean of ASVs generated from 10 samples.

Supplementary Table S3. Phylum-level microbial community during treatment of rice straw powder with rumen fluid.

	Relative abundance (%)				
	**0 h**	**12 h**	**24 h**	**36 h**	**48 h**
**Bacteria**					
Bacteroidetes	63.04	57.75	49.96	50.67	49.59
Firmicutes	18.35	15.68	15.87	17.16	20.72
Fibrobacteres	2.85	10.46	14.95	8.54	3.20
Verrucomicrobia	3.44	3.48	4.01	5.78	6.44
Spirochaetes	1.13	1.88	5.25	6.16	6.76
Tenericutes	4.01	3.81	3.83	4.20	4.50
Proteobacteria	2.53	2.89	2.39	2.88	3.60
Lentisphaerae	0.86	0.97	1.14	1.48	1.74
Cyanobacteria	1.06	0.60	0.55	0.98	1.13
SR1	0.80	0.69	0.56	0.49	0.54
Others	1.93	1.79	1.50	1.65	1.78
**Fungi**					
Neocallimastigomycota	99.99	99.90	100.00	100.00	99.99
Unclassified	0.01	0.10	0.00	0.00	0.01
**Protozoa**					
Ciliophora	100.00	100.00	100.00	100.00	100.00
Unclassified	0.00	0.00	0.00	0.00	0.00

All data represent the mean of duplicated samples.

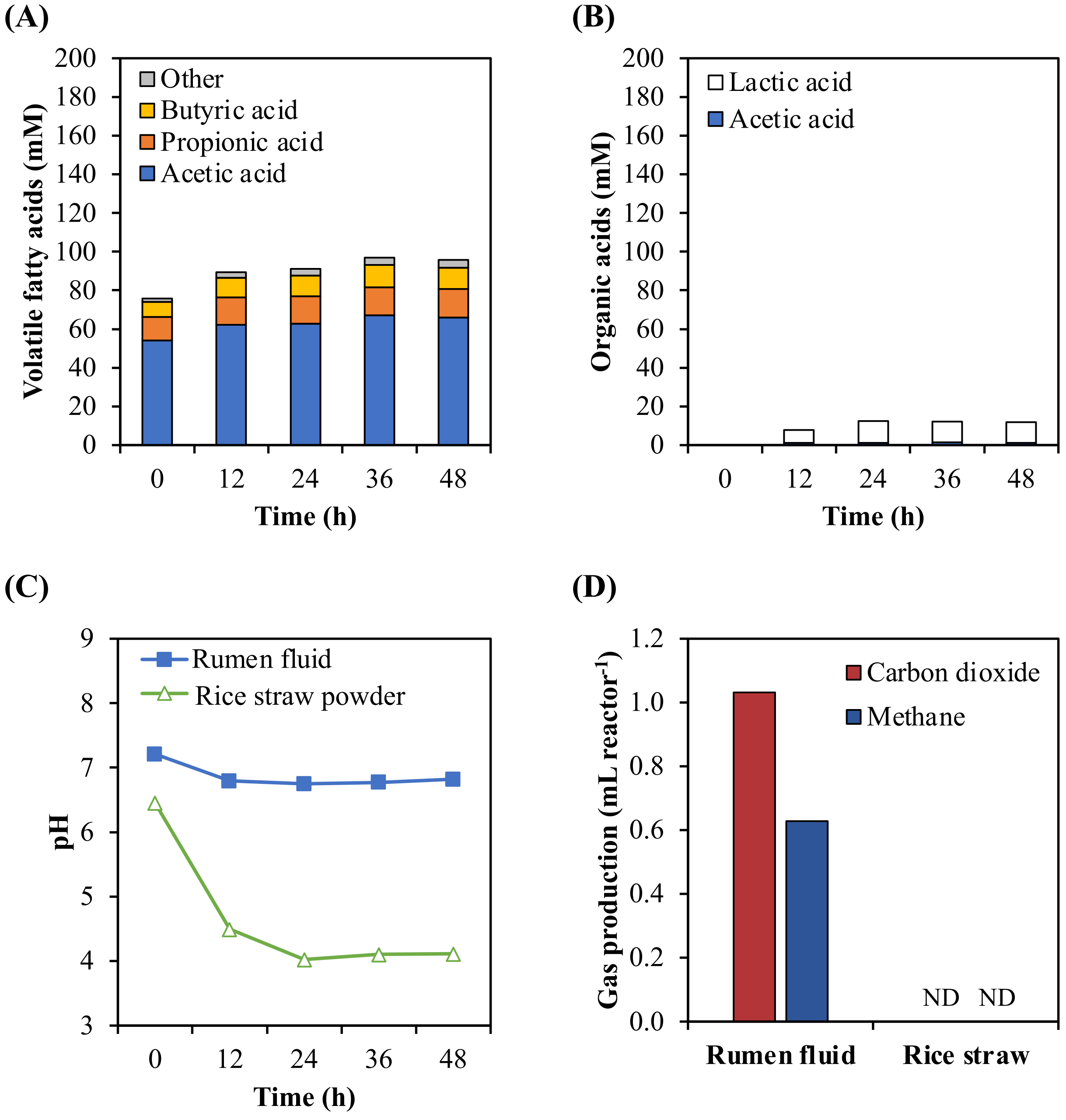

Supplementary Fig. S1. **Chemical characteristics in the blanks during the incubation for 48 h.** Organic acids concentration in the blank containing only rumen fluid (A) and only rice straw powder (B). (C) The pH values in the blanks. (D) Gas production after 48 h in the blanks. All data represent the mean of duplicated samples. ND, not detected.

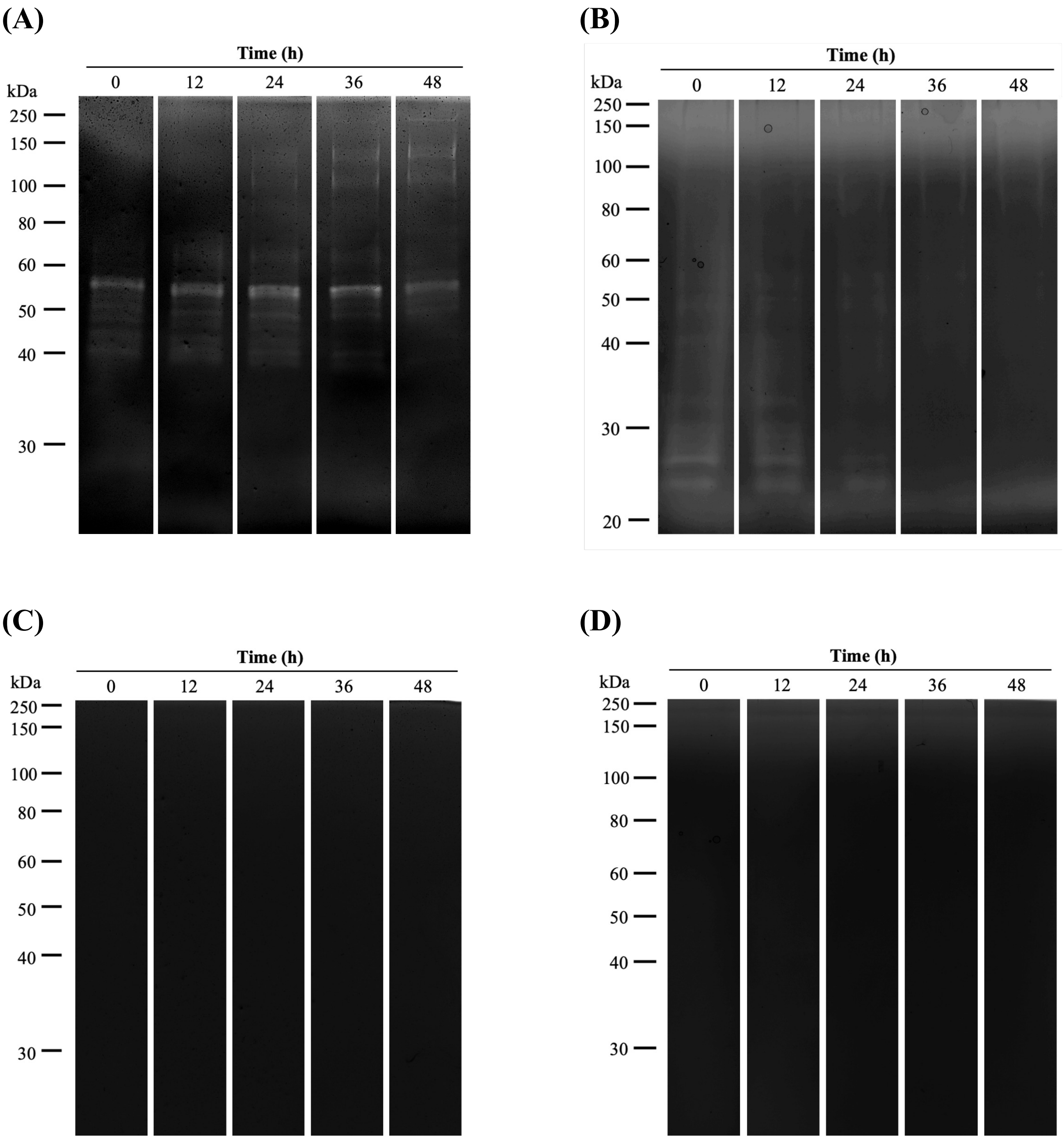

Supplementary Fig. S2. Carboxymethyl-cellulase and xylanase activities in the blank containing only rumen fluid or rice straw powder. (A) Carboxymethyl-cellulase and (B) xylanase zymogram in the blank containing only rumen fluid. (C) Carboxymethyl-cellulase and (D) xylanase zymogram in the blank containing only rice straw powder. A total of 20 µL of protein extract were loaded on 8% polyacrylamide gel containing 0.15% carboxymethyl cellulose salt and 1.0% xylan from beechwood for carboxymethyl-cellulase and xylanase zymograms, respectively. The incubations for carboxymethyl-cellulase and xylanase zymograms were performed at 37 ºC for 90 min and 4 h in sodium acetate buffer (30 mM), respectively.

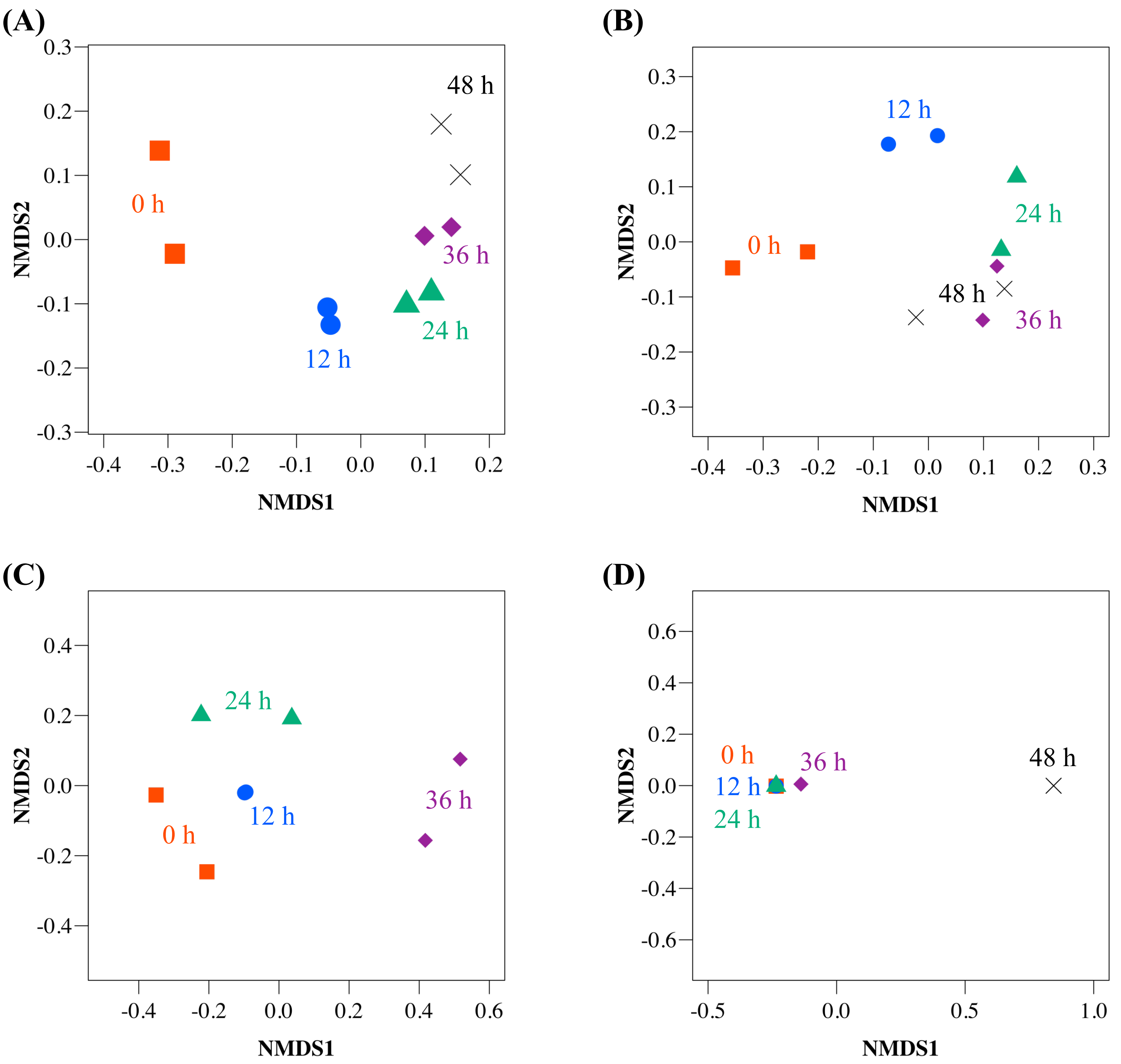

Supplementary Fig. S3. **Non-metric multidimensional scaling (NMDS) analysis of rumen microbial community.** (A) Bacterial, (B) fungal, and (C) and (D) protozoal community. (C) The structure of protozoal community during the biodegradation, except for 48 h. (D) The structure of protozoal community at 0 h, 12 h, 24 h, 36 h, and 48 h.
